# Association Between Suicidal Ideation and Cancer Screening Uptake: Results from Middle-Aged and Older Adults in Korea

**DOI:** 10.3390/cancers17060956

**Published:** 2025-03-12

**Authors:** Seong-Uk Baek, Jin-Ha Yoon

**Affiliations:** 1 Graduate School, Yonsei University College of Medicine, Seoul 03722, Republic of Korea; 2 Department of Preventive Medicine, Yonsei University College of Medicine, Seoul 03722, Republic of Korea

**Keywords:** mental health, cancer prevention, health behavior, health screening

## Abstract

Background: Compliance with cancer screening guidelines is crucial for the early diagnosis and prevention of cancer. We explored the association of suicidal ideation with participation in cancer screening programs. Methods: This cross-sectional analysis included a nationwide sample consisting of 22,554 Korean adults (9667 men and 12,887 women). Suicidal ideation in the past year was self-reported (yes or no). Participation in gastric, colorectal, cervical, and breast cancer screening within the past 2 years was assessed. Logistic regression models were employed to determine the association of suicidal ideation with participation in each cancer screening test. Odds ratios (ORs) and 95% confidence intervals (CIs) were estimated. Results: Among men, the participation rates in gastric and colorectal cancer screening were 47.6% and 39.7%, respectively. Among women, the participation rates in gastric, colorectal, cervical, and breast cancer screening were 47.1%, 34.8%, 44.3%, and 50.8%, respectively. In the male sample, suicidal ideation was associated with reduced participation in gastric cancer screening (OR: 0.83, 95% CI: 0.69–0.99) and colorectal cancer screening (OR: 0.82, 95% CI: 0.67–1.00). Similarly, in the female sample, suicidal ideation was inversely associated with participation in gastric (OR: 0.74, 95% CI: 0.67–0.82), colorectal (OR: 0.71, 95% CI: 0.62–0.81), cervical (OR: 0.75, 95% CI: 0.68–0.84), and breast cancer screening (OR: 0.76, 95% CI: 0.68–0.84). Conclusions: This study suggests that individuals with suicidal ideation demonstrated reduced participation in cancer screening tests. This study highlights the need for targeted support to improve access to cancer screening programs for individuals with mental health problems.

## Simple Summary

We examined the association of suicidal ideation with participation in cancer screening programs in a population-based sample of 22,554 adults aged ≥40 years. The main findings indicated that individuals with suicidal ideation were less likely than the general population to participate in cancer screening, including screenings for gastric, colorectal, cervical, and breast cancers. These findings suggest that individuals with suicidal ideation may encounter barriers to accessing cancer screening, underscoring the need for targeted policy interventions to enhance their participation in such programs.

## 1. Introduction

South Korea is currently experiencing rapid population aging, leading to an anticipated surge in the disease burden associated with cancer [1]. Recognizing the importance of early detection and intervention, South Korea’s National Health Insurance System implemented the National Cancer Screening Program (NCSP) in 1999, initially focusing on screenings for gastric, cervical, and breast cancers, primarily targeting middle-aged and older individuals aged 40 years and above. In 2004, screening for colorectal cancer was added to the program [2,3,4]. The National Health Insurance System covers more than 99% of the South Korean population and subsidizes 90% of the cost of the NCSP while providing free screenings for low-income individuals. Despite these efforts, participation rates in the NCSP have stagnated since 2010 [3,5], highlighting the need to identify vulnerable groups who underutilize screening services. Specifically, a recent nationwide study in Korea showed that in 2023, the participation rates for cancer screening according to the recommended NCSP guidelines were 77.5% for gastric cancer, 70.7% for colorectal cancer, 72.7% for breast cancer, and 70.2% for cervical cancer [6].

In South Korea, suicidal behavior and its related health burdens constitute significant public health concerns due to the country’s high suicide rate [7]. Suicidal behavior, particularly among middle-aged or older adults, is a significant public concern. Among middle-aged or older adults, approximately 18.3% experience suicidal ideation [8], and among individuals aged 65 and older, 43 out of every 100,000 individuals die by suicide each year [9]. Previous studies indicated that individuals with mental illness are at a higher risk of cancer-related mortality [10,11,12]. One explanation for the correlation between mental health problems and cancer mortality is the lower engagement of individuals with mental health conditions in health-promoting behaviors. For instance, such individuals are more likely to lead sedentary lifestyles, smoke, and consume alcohol, all of which increase their cancer risk [11,13]. Moreover, individuals with mental illness are less likely to undergo cancer screening, which may contribute to late cancer diagnoses and ultimately lead to high cancer mortality. For example, a study in England revealed that individuals with mental illnesses, such as schizophrenia or bipolar disorder, had a lower likelihood of participating in colorectal, breast, or cervical cancer screening [14]. Additionally, women experiencing depression had a lower likelihood of engagement in screening tests for cervical or breast cancer [15,16,17].

Various factors can influence participation in cancer screenings among individuals with suicidal ideation. For instance, individuals with poor mental health, including suicidal ideation, may have reduced motivation and interest in health screenings [18,19]. They may avoid or delay seeking necessary healthcare, which can lead to lower participation. Additionally, individuals with poor mental health often have poor socioeconomic status [20], where lower education and income levels may act as barriers to healthcare access. Moreover, individuals with suicidal ideation and poor mental health are often exposed to social stigma, which can deter them from visiting healthcare services and receiving appropriate care [21,22].

Although studies have provided meaningful insights into the relation between mental health problems and cancer screening uptake, several gaps remain in the literature. First, studies have mostly focused on the association of mental health problems with cervical or breast cancer among women [15,16,17], underscoring the necessity of including a broader range of cancer types. Second, while research has examined various mental health problems, including depression and anxiety, and their association with cancer screening participation, the relation between suicidal ideation and cancer screening uptake has been rarely studied. A study of Korean adults explored this topic but found no clear correlation between suicidal ideation and participation in any type of cancer screening [23]. However, this study did not consider cancer type or sex, highlighting the need for in-depth analyses of these factors [23]. Therefore, our research question was to explore the association of suicidal ideation with cancer screening uptake in middle-aged and older men and women in Korea.

## 2. Methods

### 2.1. Study Population

This cross-sectional study employed data derived from the Korea National Health and Nutrition Examination Survey (KNHANES) [24]. For each KNHANES wave, study participants were selected through multistage clustered probability sampling. Initially, 512 regions in Korea served as primary sampling units. Then, households were systematically selected to ensure the national representativeness of the study sample. To gather information from the participants, household visits were conducted, and surveys were administered by trained interviewers. Survey weights were assigned to all participants [25].

**Figure 1:**
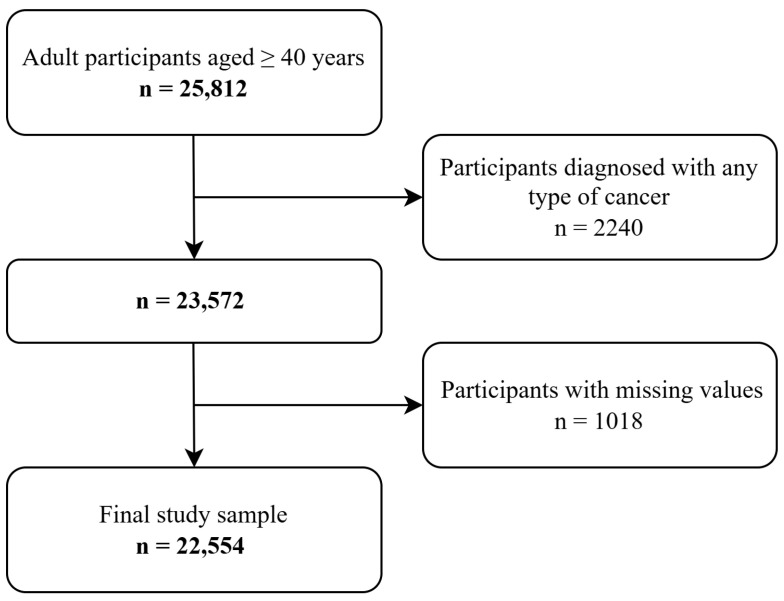
Diagram of the sample selection.

A diagram of the sample selection procedure is illustrated in Figure 1. This study included participants from the fourth and fifth waves of the KNHANES, conducted between 2007 and 2012. The rationale for this selection was that the data on detailed information regarding cancer screening by type were collected from 2007 to 2012. Since 2013, the KNHANES has not included measurements for categorizing screening participation by cancer type, preventing the use of more updated datasets. Among these participants, 25,812 middle-aged and older individuals aged ≥40 years were selected. Subsequently, those diagnosed with any type of cancer (n = 2240) and those with missing data (n = 1018) were excluded, resulting in a sample of 22,554 adults.

### 2.2. Ethical Consideration and Data Availability Statement

This secondary data analysis was approved by the Institutional Review Board of Severance Hospital, Yonsei Health System (4–2023–0959, approval date: 13 September 2023). Raw data are available at https://knhanes.kdca.go.kr (accessed on 5 February 2025). 

### 2.3. Main Variables

This study examined the participation in screening for four types of cancer: gastric, colorectal, cervical, and breast. The screening modalities and intervals were defined based on the guidelines established by the NCSP, which have been applied since 2004 [3,4]. Measurements regarding cancer screening status consisted of two questions for each of the four domains: one regarding whether the screening was performed and the other regarding the timing of the screening. This instrument has been widely used in previous nationwide epidemiological studies in Korea which examined factors associated with cancer screening participation [26,27,28,29].

Individuals who reported undergoing either an esophagogastroduodenoscopy or upper gastrointestinal series in the past 2 years were classified as having participated in gastric cancer screening. Those who reported undergoing a fecal immunochemical test or colonoscopy in the past year were classified as having participated in colorectal cancer screening. The NCSP guidelines have recommended annual colorectal screening since 2004 using fecal immunochemical testing as the primary screening method. Additionally, the NCSP offers colonoscopy screening to individuals who test positive on fecal immunochemical testing.

Among the female participants, those who reported receiving a Pap smear in the past 2 years were considered as having participated in cervical cancer screening. Women who had undergone mammography in the past 2 years were considered as having participated in breast cancer screening. For each cancer screening, adherence to the recommended guidelines was coded as “1”, while non-participation was coded as “0”.

Suicidal outcomes were determined based on a single question assessing the single domain of suicidal ideation. Suicidal ideation was determined based on the question “Have you seriously considered suicide within the past year?” Participants who answered “yes” were considered as having suicidal ideation, whereas those who answered “no” were considered as not having suicidal ideation. Individuals with suicidal ideation were coded as “1”, while those without were coded as “0”. This instrument is a standard metric employed within national, population-based surveys in the Republic of Korea, such as the Korea Health Panel Survey [30], the Korea Community Health Survey [31], and the KNHANES [32]. Additionally, this instrument has been used to investigate population-level trends in suicidal ideation and behavior in Korea [33].

### 2.4. Covariates

The following characteristics were adjusted for in the analyses: residential region: classified as urban or rural; age: treated as a continuous variable; educational level: classified as middle school or below, high school, or college and above; income level: classified into four groups—lowest, low, high, and highest—according to the quartile values of monthly household income for each survey year; marital status: classified as married, unmarried, or others (divorced, separated, or widowed); economic activity: classified as active (worker) or inactive (unemployed); chronic condition: classified as present if individuals were diagnosed with any of the following: cardiovascular disease, pulmonary disease, chronic kidney disease, diabetes mellitus, chronic viral hepatitis, or liver cirrhosis; and personal medical insurance coverage: classified as yes or no. 

### 2.5. Analysis Strategy

Descriptive analyses were performed to examine the sociodemographic features of the study sample and to calculate the percentage of participants undergoing each type of cancer screening. Logistic regression models were employed to determine the association of suicidal ideation with participation in cancer screening. The unadjusted and fully adjusted logistic regression models were sequentially fitted. Odds ratios (ORs) with 95% confidence intervals (CIs) were calculated.

For colorectal cancer screening, the sample was restricted to those aged ≥50 years, in accordance with NCSP recommendations and coverage (n = 16,268). Additionally, only female workers were included in the cervical and breast cancer screening analyses (N = 12,887).

Statistical analyses and visualizations were carried out using R software (version 4.4.1). To reflect the survey design, the R package “survey” and its function “svyglm” were used to conduct logistic regressions that incorporated survey weights [34]. 

## 3. Results

The sample consisted of 9667 men and 12,887 women (Table 1). The prevalence of suicidal ideation was 11.7% among men and 21.4% among women. In both the male and female samples, those with suicidal ideation were more likely to live in rural regions, be older, have lower income and education levels, be unemployed, lack personal medical insurance, and have chronic conditions.

**Figure 2:**
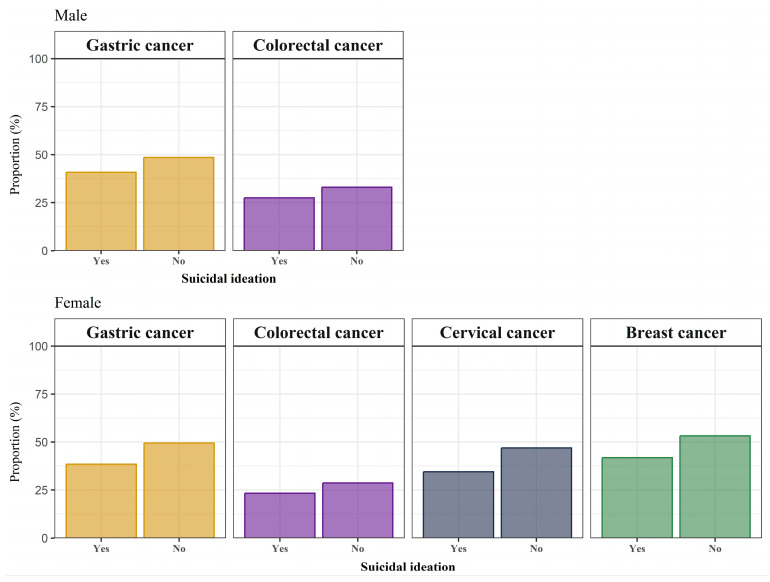
Prevalence of uptake of cancer screening programs in Korean men and women.

Figure 2 shows the screening uptake rates according to the cancer type and suicidal ideation. Among men, the overall rates of participation in gastric and colorectal cancer screening were 47.6% and 39.7%, respectively. Among women, the overall rates of participation in gastric, colorectal, cervical, and breast cancer screening were 47.1%, 34.8%, 44.3%, and 50.8%, respectively. For all cancer types, the cancer screening participation rate was lower among those with suicidal ideation than among those without.

Table 2 shows the factors associated with participation in gastric and colorectal cancer screening programs among the male participants. Age was positively associated with uptake of the screening program for gastric cancer, whereas low income levels, low educational levels, being unmarried, and lacking personal medical insurance were negatively associated with the uptake of the screening program for gastric cancer. Suicidal ideation was negatively associated with uptake of gastric cancer screening (OR: 0.83, 95% CI: 0.69–0.99). Similarly, age was positively associated with uptake of the screening program for colorectal cancer, whereas low income levels, low educational levels, being unmarried, and lacking personal medical insurance were negatively associated with uptake of the screening program for colorectal cancer. Suicidal ideation was marginally negatively associated with uptake of colorectal cancer screening (OR: 0.82, 95% CI: 0.67–1.00).

Table 3 shows the factors related to engagement in gastric and colorectal cancer screening programs among female participants. Age was positively associated with uptake of the screening program for gastric cancer, whereas low income levels, being unmarried, and lacking personal medical insurance were negatively associated with uptake of the screening program for gastric cancer. Suicidal ideation was negatively associated with uptake of gastric cancer screening (OR: 0.74, 95% CI: 0.67–0.82). Additionally, residing in rural regions, age, an unmarried status, and lacking personal medical insurance were negatively correlated with uptake of colorectal cancer screening. Suicidal ideation was marginally negatively correlated with uptake of colorectal cancer screening (OR: 0.71, 95% CI: 0.62–0.81).

Table 4 shows the factors related to engagement in cervical and breast cancer screening programs among female participants. Residing in rural regions, age, low income levels, being unmarried, and lacking personal medical insurance were negatively associated with uptake of the screening program for cervical cancer. Suicidal ideation was negatively associated with uptake of cervical cancer screening (OR: 0.75, 95% CI: 0.68–0.84). Additionally, low income levels, being unmarried, and lacking personal medical insurance were negatively associated with uptake of the screening program for breast cancer. Suicidal ideation was negatively associated with uptake of breast cancer screening (OR: 0.76, 95% CI: 0.68–0.84). 

## 4. Discussion

This study found that suicidal ideation was negatively associated with the likelihood of participating in gastric and colorectal cancer screenings in both men and women. Moreover, women with suicidal ideation were less likely to undergo cervical and breast cancer screening than those without suicidal ideation. Consequently, this study highlights the need for policy interventions to promote cancer screening among individuals with suicidal ideation.

These findings are in line with those of prior research indicating that those with poor mental health are less likely to engage in cancer screening. For instance, one meta-analysis showed that individuals with mental illnesses were less likely to undergo screening tests for breast, cervical, and prostate cancers [35]. A largescale cross-sectional study of British adults showed that those with severe mental illness had 0.51-fold, 0.61-fold, and 0.78-fold lower odds of participating in colorectal, breast, and cervical cancer screening within the recommended time period, respectively [14]. Furthermore, research has consistently shown that individuals experiencing depressive symptoms exhibit a diminished likelihood of engaging in screening tests for cervical or breast cancer [15,16,17]. By contrast, a study on Korean adults found no clear association between suicidal ideation and engagement in screening tests for any type of cancer after adjusting for the sociodemographic characteristics of the survey participants [23]. However, the previous Korean study included a smaller sample size than that in the present study and did not stratify the analysis by cancer type or sex, limiting the understanding of a more nuanced relation between suicidal ideation and the underuse of cancer screening [23]. Consequently, compared with previous research, this study adds to the body of literature by revealing a negative association between suicidal ideation and screening for various cancer types.

Various factors can influence individuals’ participation in healthcare. For instance, the Self-Perception Theory of Health and Wellness posits that an individual’s self-perception of health influences their health behaviors [36]. Complex mechanisms may underpin the relation between suicidal ideation and the reduced likelihood of cancer screening. First, individuals with suicidal ideation may have reduced motivation and energy to engage in healthcare services, including cancer screenings [18,19]. Additionally, those with suicidal ideation may experience reduced social functioning, making it difficult for them to schedule appointments and participate in screenings [37]. Suicidal ideation is often associated with psychiatric comorbidities [38], which are characterized by psychomotor retardation, diminished interest, and cognitive impairments. These factors contribute to the reluctance of individuals with suicidal ideation to undergo cancer screening. Second, individuals with suicidal ideation are more likely to experience social stigma and a lack of support, which can hinder their participation in cancer screening. For instance, healthcare providers may hold misconceptions or negative stereotypes about patients with suicidal thoughts or mental illnesses [21,22], which can negatively affect the rapport between patients and providers. This, in turn, can lead to the underuse of preventive healthcare services, including cancer screening. Third, negative perceptions and beliefs about healthcare services may significantly influence the likelihood of screening participation among individuals experiencing suicidal ideation. For example, individuals with mental health problems are more likely to hold negative attitudes toward breast cancer screening, which may contribute to lower participation rates compared with those of the general population [39,40].

This study has several policy implications. First, the findings indicate that individuals with suicidal ideation are less likely to participate in cancer screenings. Therefore, in clinical settings, primary healthcare providers should closely monitor their adherence to recommended cancer screening guidelines and encourage their participation. Additionally, policy efforts should focus on identifying potential barriers to cancer screening uptake among individuals with suicidal ideation—such as reduced motivation, stigma, lack of support, and negative attitudes—and on developing targeted strategies to address these challenges.

Some limitations should be noted. First, our analyses were based on cross-sectional analyses; thus, a causal relation between suicidal ideation and the uptake of cancer screening cannot be asserted. There is a possibility that reverse causation should be considered, wherein non-engagement in cancer screening programs might contribute to the deterioration of the mental health of the study participants. Therefore, future longitudinal studies should be conducted to elucidate whether suicidal ideation can predict engagement in cancer screening programs. Second, engagement in cancer screening programs was assessed via self-reporting, which may cause measurement errors, including recall bias. Additionally, the validity and reliability of the self-reported measurements used in this study have not been reported, which limits the robustness of the measurements used in this study. Therefore, future research can benefit from employing more objective methods, such as the use of medical records, to ensure the accuracy of the variables. Third, suicidal ideation was assessed using a single-item questionnaire, which may have introduced recall and misclassification bias. The lack of reported validity for this measurement compared to multi-item questions represents a limitation of this study. Future studies should consider using a validated multi-item questionnaire to enhance measurement reliability. [41]. Fourth, as this study employed data collected up to 2012, owing to the lack of information, the authors recognize the necessity for future research to utilize more updated data. Nevertheless, given that the screening methodologies and eligibility criteria for cancer screening within the Korean NCSP have remained consistent [3,4], the results of this study offer valuable insights within the current context. Fifth, the association between suicidal ideation and the underuse of cancer screening was more pronounced in women. It should be considered that the association between suicidal ideation and participation in gastric and colorectal cancer screening in men was modest and may not be clinically meaningful.

## 5. Conclusions

This study found that suicidal ideation was linked to a reduced likelihood of engaging in cancer screening programs among middle-aged and older Korean adults. A negative association was observed between suicidal ideation and participation in cancer screening in both men and women. Therefore, our study suggests the need for policy support to promote cancer screening programs for individuals with poor mental health.

## Figures and Tables

**Table 1:** Characteristics of the study sample.

	Male		Female	
	Suicidal Ideation		Suicidal Ideation	
	Yes	No	Yes	No
	N = 1194	N = 8473	N = 2845	N = 10,042
Region				
Urban	799 (66.9)	6098 (72.0)	1899 (66.7)	7344 (73.1)
Rural	395 (33.1)	2375 (28.0)	946 (33.3)	2698 (26.9)
Age				
Median (Q1, Q3)	62.0 (51.0, 71.0)	57.0 (48.0, 67.0)	64.0 (52.0, 72.0)	56.0 (48.0, 67.0)
Income level				
Highest	199 (16.7)	2412 (28.5)	401 (14.1)	2678 (26.7)
High	213 (17.8)	2197 (25.9)	510 (17.9)	2341 (23.3)
Low	339 (28.4)	2107 (24.9)	737 (25.9)	2493 (24.8)
Lowest	443 (37.1)	1757 (20.7)	1197 (42.1)	2530 (25.2)
Education level				
College or above	194 (16.2)	2280 (26.9)	179 (6.3)	1349 (13.4)
High school	276 (23.1)	2712 (32.0)	477 (16.8)	2789 (27.8)
Middle school or below	724 (60.6)	3481 (41.1)	2189 (76.9)	5904 (58.8)
Employment status				
Active	720 (60.3)	6359 (75.1)	1154 (40.6)	4832 (48.1)
Inactive	474 (39.7)	2114 (24.9)	1691 (59.4)	5210 (51.9)
Marital status				
Married	993 (83.2)	7842 (92.6)	1754 (61.7)	7478 (74.5)
Never married	43 (3.6)	150 (1.8)	29 (1.0)	91 (0.9)
Others	158 (13.2)	481 (5.7)	1062 (37.3)	2473 (24.6)
Personal medical insurance				
Yes	553 (46.3)	5405 (63.8)	1352 (47.5)	6607 (65.8)
No	641 (53.7)	3068 (36.2)	1493 (52.5)	3435 (34.2)
Chronic condition				
No	861 (72.1)	6910 (81.6)	2173 (76.4)	8656 (86.2)
Yes	333 (27.9)	1563 (18.4)	672 (23.6)	1386 (13.8)

Values are presented as n (%).

**Table 2:** Factors related to participation in gastric and colorectal cancer screenings among men.

	Gastric Cancer		Colorectal Cancer	
	Model 1	Model 2	Model 1	Model 2
	OR (95% CI)	OR (95% CI)	OR (95% CI)	OR (95% CI)
Suicidal ideation				
No	Reference	Reference	Reference	Reference
Yes	0.73 (0.62–0.87)	0.83 (0.69–0.99)	0.71 (0.58–0.86)	0.82 (0.67–1.00)
Region				
Urban		Reference		Reference
Rural		1.04 (0.91–1.18)		0.90 (0.77–1.05)
Age				
Continuous scale		1.03 (1.02–1.03)		1.02 (1.01–1.03)
Income level				
Highest		Reference		Reference
High		0.76 (0.66–0.86)		0.87 (0.72–1.05)
Low		0.74 (0.63–0.87)		0.90 (0.74–1.09)
Lowest		0.61 (0.51–0.73)		0.62 (0.50–0.76)
Education level				
College or above		Reference		Reference
High school		0.74 (0.65–0.84)		0.85 (0.70–1.03)
Middle school or below		0.75 (0.64–0.87)		0.74 (0.61–0.90)
Employment status				
Active		Reference		Reference
Inactive		0.89 (0.77–1.03)		0.94 (0.80–1.09)
Marital status				
Married		Reference		Reference
Never married		0.82 (0.57–1.17)		0.86 (0.44–1.71)
Others		0.67 (0.54–0.83)		0.64 (0.49–0.84)
Personal medical insurance				
Yes		Reference		Reference
No		0.61 (0.53–0.71)		0.74 (0.64–0.87)
Chronic condition				
No		Reference		Reference
Yes		1.00 (0.88–1.14)		1.02 (0.89–1.18)

OR, odds ratio; CI, confidence interval.

**Table 3:** Factors related to participation in gastric and colorectal cancer screenings among women.

	Gastric Cancer		Colorectal Cancer	
	Model 1	Model 2	Model 1	Model 2
	OR (95% CI)	OR (95% CI)	OR (95% CI)	OR (95% CI)
Suicidal ideation				
No	Reference	Reference	Reference	Reference
Yes	0.64 (0.58–0.70)	0.74 (0.67–0.82)	0.60 (0.52–0.68)	0.71 (0.62–0.81)
Region				
Urban		Reference		Reference
Rural		1.06 (0.94–1.19)		0.75 (0.64–0.89)
Age				
Continuous scale		1.01 (1.00–1.02)		0.99 (0.98–0.99)
Income level				
Highest		Reference		Reference
High		0.83 (0.73–0.95)		1.13 (0.95–1.34)
Low		0.79 (0.69–0.90)		1.07 (0.91–1.27)
Lowest		0.75 (0.65–0.87)		1.03 (0.87–1.21)
Education level				
College or above		Reference		Reference
High school		0.95 (0.82–1.10)		1.03 (0.80–1.34)
Middle school or below		0.96 (0.82–1.13)		0.94 (0.73–1.22)
Employment status				
Active		Reference		Reference
Inactive		0.97 (0.88–1.07)		1.02 (0.90–1.16)
Marital status				
Married		Reference		Reference
Never married		0.75 (0.47–1.20)		0.88 (0.46–1.70)
Others		0.72 (0.64–0.80)		0.81 (0.70–0.92)
Personal medical insurance				
Yes		Reference		Reference
No		0.51 (0.45–0.58)		0.61 (0.53–0.70)
Chronic condition				
No		Reference		Reference
Yes		1.07 (0.95–1.21)		1.07 (0.93–1.22)

OR, odds ratio; CI, confidence interval.

**Table 4:** Factors related to participation in cervical and breast cancer screenings among women.

	Cervical Cancer		Breast Cancer	
	Model 1	Model 2	Model 1	Model 2
	OR (95% CI)	OR (95% CI)	OR (95% CI)	OR (95% CI)
Suicidal ideation				
No	Reference	Reference	Reference	Reference
Yes	0.60 (0.53–0.66)	0.75 (0.68–0.84)	0.63 (0.57–0.70)	0.76 (0.68–0.84)
Region				
Urban		Reference		Reference
Rural		0.75 (0.66–0.85)		1.06 (0.94–1.19)
Age				
Continuous scale		0.98 (0.98–0.99)		1.00 (0.99–1.01)
Income level				
Highest		Reference		Reference
High		0.85 (0.74–0.96)		0.85 (0.74–0.98)
Low		0.90 (0.78–1.02)		0.81 (0.70–0.93)
Lowest		0.86 (0.75–1.00)		0.86 (0.74–0.99)
Education level				
College or above		Reference		Reference
High school		0.89 (0.76–1.04)		0.91 (0.78–1.06)
Middle school or below		0.89 (0.75–1.05)		0.89 (0.76–1.04)
Employment status				
Active		Reference		Reference
Inactive		1.00 (0.90–1.11)		0.93 (0.84–1.03)
Marital status				
Married		Reference		Reference
Never married		0.67 (0.41–1.08)		0.60 (0.38–0.95)
Others		0.79 (0.70–0.88)		0.79 (0.71–0.88)
Personal medical insurance				
Yes		Reference		Reference
No		0.55 (0.48–0.62)		0.52 (0.46–0.58)
Chronic condition				
No		Reference		Reference
Yes		0.94 (0.83–1.07)		1.01 (0.89–1.14)

OR, odds ratio; CI, confidence interval.

## Data Availability

Raw data are available at https://knhanes.kdca.go.kr (accessed on 5 February 2025).

## References

[1] GBD South Korea BoD Collaborators (2023). Population health outcomes in South Korea 1990–2019, and projections up to 2040: A systematic analysis for the Global Burden of Disease Study 2019. Lancet Public Health.

[2] Shin D.W., Cho J., Park J.H., Cho B. (2022). National General Health Screening Program in Korea: History, current status, and future direction. Precis. Future Med..

[3] Kang H.T. (2022). Current Status of the National Health Screening Programs in South Korea. Korean J. Fam. Med..

[4] Kim Y., Jun J.K., Choi K.S., Lee H.Y., Park E.C. (2011). Overview of the National Cancer screening programme and the cancer screening status in Korea. Asian Pac. J. Cancer Prev..

[5] Park B., Her E.Y., Lee K., Nari F., Jun J.K., Choi K.S., Suh M. (2023). Overview of the National Cancer Screening Program for Colorectal Cancer in Korea over 14 Years (2004–2017). Cancer Res. Treat..

[6] Kang E., Choi K.S., Jun J.K., Kim Y., Lee H.J., Choi C.K., Kim T.H., Lee S.H., Suh M. (2025). Trends in Cancer-Screening Rates in Korea: Findings from the National Cancer Screening Survey, 2004–2023. Cancer Res. Treat..

[7] Hong M., Kim H., Park C.H.K., Lee H., Rhee S.J., Min S., Kim M.J., Yang J.H., Song Y., Son K. (2024). Effect of community attitudes on suicide mortality in South Korea: A nationwide ecological study. Front. Psychiatry.

[8] Choi Y.J., Lee W.Y. (2017). The prevalence of suicidal ideation and depression among primary care patients and current management in South Korea. Int. J. Ment. Health Syst..

[9] Kim E., Kim S. (2024). Spatially clustered patterns of suicide mortality rates in South Korea: A geographically weighted regression analysis. BMC Public. Health.

[10] Musuuza J.S., Sherman M.E., Knudsen K.J., Sweeney H.A., Tyler C.V., Koroukian S.M. (2013). Analyzing excess mortality from cancer among individuals with mental illness. Cancer.

[11] Gilham K., Gadermann A., Dummer T., Murphy R.A. (2023). Mental health, cancer risk, and the mediating role of lifestyle factors in the CARTaGENE cohort study. PLoS ONE.

[12] Chierzi F., Stivanello E., Musti M.A., Perlangeli V., Marzaroli P., De Rossi F., Pandolfi P., Saponaro A., Grassi L., Belvederi Murri M. (2023). Cancer mortality in Common Mental Disorders: A 10-year retrospective cohort study. Soc. Psychiatry Psychiatr. Epidemiol..

[13] Pan K.Y., van Tuijl L., Basten M., Rijnhart J.J.M., de Graeff A., Dekker J., Geerlings M.I., Hoogendoorn A., Ranchor A.V., Vermeulen R. (2024). The mediating role of health behaviors in the association between depression, anxiety and cancer incidence: An individual participant data meta-analysis. Psychol. Med..

[14] Kerrison R.S., Jones A., Peng J., Price G., Verne J., Barley E.A., Lugton C. (2023). Inequalities in cancer screening participation between adults with and without severe mental illness: Results from a cross-sectional analysis of primary care data on English Screening Programmes. Br. J. Cancer.

[15] Kaida A., Colman I., Janssen P.A. (2008). Recent Pap tests among Canadian women: Is depression a barrier to cervical cancer screening?. J. Womens Health Larchmt..

[16] Vigod S.N., Kurdyak P.A., Stewart D.E., Gnam W.H., Goering P.N. (2011). Depressive symptoms as a determinant of breast and cervical cancer screening in women: A population-based study in Ontario, Canada. Arch. Womens Ment. Health.

[17] Burato S., D’Aietti A., Paci A., Pellegrini L., Di Salvo G., Sindici C., Dellach C., Negro S., Albert U. (2025). Elevated mortality risks associated with late diagnosis of cancer in individuals with psychiatric disorders?. J. Psychiatr. Res..

[18] Kelsey E.A., West C.P., Cipriano P.F., Peterson C., Satele D., Shanafelt T., Dyrbye L.N. (2021). Original Research: Suicidal Ideation and Attitudes Toward Help Seeking in U.S. Nurses Relative to the General Working Population. Am. J. Nurs..

[19] Loas G., Lefebvre G., Rotsaert M., Englert Y. (2018). Relationships between anhedonia, suicidal ideation and suicide attempts in a large sample of physicians. PLoS ONE.

[20] Ju Y.J., Park E.C., Han K.T., Choi J.W., Kim J.L., Cho K.H., Park S. (2016). Low socioeconomic status and suicidal ideation among elderly individuals. Int. Psychogeriatr..

[21] Jandial R., Subramanian K., Kumar S., Subramanian E., Balasundaram S. (2024). Literacy and Attitude Toward Suicide Among Doctors and Nurses: A Cross-Sectional Comparative Study. Cureus.

[22] Rimkeviciene J., Hawgood J., O’Gorman J., De Leo D. (2015). Personal Stigma in Suicide Attempters. Death Stud..

[23] Ki M., Shim H.Y., Lim J., Hwang M., Kang J., Na K.S. (2022). Preventive health behaviors among people with suicide ideation using nationwide cross-sectional data in South Korea. Sci. Rep..

[24] Kweon S., Kim Y., Jang M.J., Kim Y., Kim K., Choi S., Chun C., Khang Y.H., Oh K. (2014). Data resource profile: The Korea National Health and Nutrition Examination Survey (KNHANES). Int. J. Epidemiol..

[25] Oh K., Kim Y., Kweon S., Kim S., Yun S., Park S., Lee Y.K., Kim Y., Park O., Jeong E.K. (2021). Korea National Health and Nutrition Examination Survey, 20th anniversary: Accomplishments and future directions. Epidemiol. Health.

[26] Chuck K.W., Hwang M., Choi K.S., Suh M., Jun J.K., Park B. (2017). Cancer screening rate in people with diabetes in the Korean population: Results from the Korea National Health and Nutrition Examination Survey 2007–2009. Epidemiol. Health.

[27] Kim E.Y., Shim Y.S., Kim Y.S., Lee S.P., Ko K.D., Choi W.J. (2019). Adherence to general medical checkup and cancer screening guidelines according to self-reported smoking status: Korea National Health and Nutrition Examination Survey (KNHANES) 2010–2012. PLoS ONE.

[28] Kang M., Yoo K.B., Park E.C., Kwon K., Kim G., Kim D.R., Kwon J.A. (2014). Factors associated with organized and opportunistic cancer screening: Results of the Korea National Health and Nutrition Examination Survey (KNHANES) 2007-2011. Asian Pac. J. Cancer Prev..

[29] Myong J.P., Shin J.Y., Kim S.J. (2012). Factors associated with participation in colorectal cancer screening in Korea: The Fourth Korean National Health and Nutrition Examination Survey (KNHANES IV). Int. J. Color. Dis..

[30] Kim S.M., Lee G. (2020). Risk factors of suicide ideation in younger-old and older-old persons: Using data from the korea health panel survey. J. Korean Gerontol. Nurs..

[31] Hwang I.C., Ahn H.Y. (2021). Association between neighborhood environments and suicidal ideation among Korean adults. J. Affect. Disord..

[32] Han K.M., Won E., Paik J.W., Lee M.S., Lee H.W., Ham B.J. (2016). Mental health service use in adults with suicidal ideation within a nationally representative sample of the Korean population. J. Affect. Disord..

[33] Kim S.H., Lee D.W., Kwon J., Yang J., Park E.C., Jang S.I. (2021). Suicide related indicators and trends in Korea in 2019. Health Policy Manag..

[34] Lumley T. (2004). Analysis of complex survey samples. J. Stat. Softw..

[35] Solmi M., Firth J., Miola A., Fornaro M., Frison E., Fusar-Poli P., Dragioti E., Shin J.I., Carvalho A.F., Stubbs B. (2020). Disparities in cancer screening in people with mental illness across the world versus the general population: Prevalence and comparative meta-analysis including 4 717 839 people. Lancet Psychiatry.

[36] Rano-Santamaria O., Fernandez-Merino C., Castano-Carou A.I., Lado-Baleato O., Fernandez-Dominguez M.J., Sanchez-Castro J.J., Gude F. (2022). Health self-perception is associated with life-styles and comorbidities and its effect on mortality is confounded by age. A Popul. Based Study. Front. Med. Lausanne.

[37] Szanto K., Dombrovski A.Y., Sahakian B.J., Mulsant B.H., Houck P.R., Reynolds C.F., Clark L. (2012). Social emotion recognition, social functioning, and attempted suicide in late-life depression. Am. J. Geriatr. Psychiatry.

[38] Sanchez-Carro Y., de la Torre-Luque A., Diaz-Marsa M., Aguayo-Estremera R., Andreo-Jover J., Ayad-Ahmed W., Bobes J., Bobes-Bascaran T., Bravo-Ortiz M.F., Canal-Rivero M. (2024). Psychiatric profiles in suicidal attempters: Relationships with suicide behaviour features. Span. J. Psychiatry Ment. Health.

[39] Park C., Ma X., Park S.K., Lawson K.A. (2020). Association of depression with adherence to breast cancer screening among women aged 50 to 74 years in the United States. J. Eval. Clin. Pract..

[40] Yazgan I., Chagpar A. (2022). The effect of emotional disorders on adherence to mammography screening guidelines. Breast Cancer Res. Treat..

[41] Millner A.J., Lee M.D., Nock M.K. (2015). Single-Item Measurement of Suicidal Behaviors: Validity and Consequences of Misclassification. PLoS ONE.

